# Changes On Mean Systemic Filling Pressure And Microcirculation After a Fluid Challenge

**DOI:** 10.1186/2197-425X-3-S1-A20

**Published:** 2015-10-01

**Authors:** HD Aya, A Carsetti, S Pierantozzi, J Mellinghof, N Fletcher, A Rhodes, M Cecconi

**Affiliations:** St George's University Hospitals NHS Foundation Trust, Adult Critical Care, London, United Kingdom; St George's University of London, Cardiovascular Institute, London, United Kingdom

## Introduction

In a previous study we observed that a fluid challenge of 4 ml/Kg is adequate to raise the mean systemic filling pressure (Pmsf) and to increase venous return in fluid responsive patients.

## Objectives

The objective of this study is to describe the changes on microcirculation after a fluid challenge and describe the relationship with the changes on Pmsf. Our hypothesis is that changes in Pmsf could induce changes on the number of perfused vessels (PVD).

## Methods

24 post-operative cardiac patients were prospectively enrolled. Arterial pressure and central venous pressure was measured invasively. Cardiac output (CO) was measured with a non-calibrated LiDCO*plus* monitor (LiDCO, Cambridge). Pmsf was measured using the stop-flow arterial venous equilibrium pressure method (1). Sublingual microcirculation was recorded with CytoCam (Braedius Medical, Amsterdam, The Netherlands). Data was recorded at baseline, immediately after the fluid infusion, 5 and 10 minutes after the end of 4 ml/Kg of crystalloids infused in 5 minutes. At each time at least three sublingual microcirculation videos were acquired to assess total vessel density (TVD), microcirculatory flow index (MFI), proportion of perfused vessel (PPV), perfused vessel density (PVD) and microcirculatory heterogeneity index (MHI). Analysis was performed with AVA software v. 3.2 (MicroVision Medical, Amsterdam, The Netherlands). Responders are defined by an increase in CO greater than 10%.

## Results

Changes on haemodynamics and microcirculatory indices are presented in table 1 and 2. Pmsf increased in both groups. There were no significant changes in microcirculatory parameters, either in responders or non-responders. There was no significant correlation between changes on PVD and changes in CO (t = 0.08, *p*= 0.59) or Pmsf (t = 0.12, *p*= 0.41).

**Table 1:** Changes on haemodynamics.

	Responders (n = 14)			Non-Responders (n = 10)		
	Baseline	Peak value Post-FC	p	Baseline	Peak value Post-FC	p
SV (ml)	78.5 (63.3 - 91.3)	94.5 (80.3 - 102 .8)	0.001	69.5 (57 - 87.5)	75.5 (56.5 - 86.8)	0.1
Pmsf (mmHg)	23 (16.5 - 26)	28 (20.8 - 29)	0.01	25 (20.8 - 27.5)	26.5 (23.8 - 32)	0.04
HR (bpm)	83 (77.5 - 89.3)	84 (79.3 - 89.3)	0.25	80 (74.3 - 86.5)	80 (74 - 91)	0.20
CVP (mmHg)	9 (7.8 - 11)	10.5 (7 - 12.3)	0.24	11.5 (8.5 - 13.5)	13.5 (10.3 - 15)	0.04
MAP (mmHg)	72 (67.8 - 84.8)	86.5 (80.8 - 94)	0.001	78 (69.5 - 80.3)	80 (76.3 - 83.5)	0.03

**Table 2:** Changes on microcirculatory indices.

	Responders (n = 14)			Non-Responders (n = 10)		
	Baseline	Peak value Post-FC	p	Baseline	Peak value Post-FC	p
MFI	2.83 (2.66 - 3)	2.83 (2.5 - 3)	0.75	2.92 (2.56 - 3)	2.85 (2.35 - 2.95)	0.21
TVD (n/mm)	10.8 (9 - 12.6)	12 (10.2 - 12.9)	0.3	12.6 (11.4 - 13)	12.1 (9.5 - 14.4)	0.79
PVD (n/mm)	9.9 (8.8 - 12.4)	11.6 (9.9 - 12.6)	0.55	12 (10.8 - 12.7)	11.2 (8.7 - 14)	0.39
PPV (%)	98.3 (94.8 - 99.2)	95.6 (90-6 - 99.1)	0.47	95.5 (94.6 - 97.4)	92.8 (89.2 - 98.7)	0.17
MHI	8.8 (0 - 34)	12.2 (0 - 28)	0.86	8.8 (0 - 25.8)	6.9 (0 - 23.5)	0.48

## Conclusions

Despite the changes in Pmsf and macrohaemodynamics, a fluid challenge does not improve microcirculation in patients after cardiac surgery.
